# Comparing Benefits from Many Possible Computed Tomography Lung Cancer Screening Programs: Extrapolating from the National Lung Screening Trial Using Comparative Modeling

**DOI:** 10.1371/journal.pone.0099978

**Published:** 2014-06-30

**Authors:** Pamela M. McMahon, Rafael Meza, Sylvia K. Plevritis, William C. Black, C. Martin Tammemagi, Ayca Erdogan, Kevin ten Haaf, William Hazelton, Theodore R. Holford, Jihyoun Jeon, Lauren Clarke, Chung Yin Kong, Sung Eun Choi, Vidit N. Munshi, Summer S. Han, Joost van Rosmalen, Paul F. Pinsky, Suresh Moolgavkar, Harry J. de Koning, Eric J. Feuer

**Affiliations:** 1 Institute for Technology Assessment, Massachusetts General Hospital, Boston, Massachusetts, United States of America; 2 Department of Radiology, Harvard Medical School, Boston, Massachusetts, United States of America; 3 Department of Epidemiology, University of Michigan, Ann Arbor, Michigan, United States of America; 4 Department of Radiology, Stanford University, Stanford, California, United States of America; 5 Department of Radiology, Dartmouth Medical School, Hanover, New Hampshire, United States of America; 6 Department of Community Health Sciences, Brock University, Ontario, Canada; 7 Department of Public Health, Erasmus MC, Rotterdam, Netherlands; 8 Program of Computational Biology, Fred Hutchinson Cancer Research Center, Seattle, Washington, United States of America; 9 Department of Biostatistics, Yale School of Public Health, New Haven, Connecticut, United States of America; 10 Department of Biostatistics and Biomathematics, Fred Hutchinson Cancer Research Center, Seattle, Washington, United States of America; 11 Cornerstone Systems Northwest, Inc., Lynden, Washington, United States of America; 12 Division of Cancer Prevention, National Cancer Institute, Bethesda, Maryland, United States of America; 13 Department of Epidemiology, School of Public Health University of Washington, Seattle, Washington, United States of America, and Department of Biostatistics and Biomathematics, Fred Hutchinson Cancer Research Center, Seattle, Washington, United States of America; 14 Division of Cancer Control and Population Sciences, National Cancer Institute, Bethesda, Maryland, United States of America; Clinica Universidad de Navarra, Spain

## Abstract

**Background:**

The National Lung Screening Trial (NLST) demonstrated that in current and former smokers aged 55 to 74 years, with at least 30 pack-years of cigarette smoking history and who had quit smoking no more than 15 years ago, 3 annual computed tomography (CT) screens reduced lung cancer-specific mortality by 20% relative to 3 annual chest X-ray screens. We compared the benefits achievable with 576 lung cancer screening programs that varied CT screen number and frequency, ages of screening, and eligibility based on smoking.

**Methods and Findings:**

We used five independent microsimulation models with lung cancer natural history parameters previously calibrated to the NLST to simulate life histories of the US cohort born in 1950 under all 576 programs. ‘Efficient’ (within model) programs prevented the greatest number of lung cancer deaths, compared to no screening, for a given number of CT screens. Among 120 ‘consensus efficient’ (identified as efficient across models) programs, the average starting age was 55 years, the stopping age was 80 or 85 years, the average minimum pack-years was 27, and the maximum years since quitting was 20. Among consensus efficient programs, 11% to 40% of the cohort was screened, and 153 to 846 lung cancer deaths were averted per 100,000 people. In all models, annual screening based on age and smoking eligibility in NLST was not efficient; continuing screening to age 80 or 85 years was more efficient.

**Conclusions:**

Consensus results from five models identified a set of efficient screening programs that include annual CT lung cancer screening using criteria like NLST eligibility but extended to older ages. Guidelines for screening should also consider harms of screening and individual patient characteristics.

## Introduction

In the National Lung Screening Trial (NLST) [1], participants aged 55–74 years randomized to three annual CT examinations experienced a 20% reduction in lung cancer mortality at 6.5 years of follow up (16% at 7.5 years) [2], compared to participants randomized to receive three annual chest radiographs. The NLST was designed to determine the efficacy of CT screening, but the eligibility criteria and the number of screens offered were not meant to represent a population screening strategy. Multiple clinical guidelines, however, recommend lung cancer screening for individuals meeting the NLST eligibility criteria [3], [4]. Other guidelines expanded recommendations for screening to individuals who would have been ineligible for the NLST [5]–.

The NLST provided no direct evidence of further reductions in lung cancer mortality from additional screens, or of potential benefits of screening individuals with lighter smoking histories (fewer than 30 pack-years of cigarette smoking or former smokers who had quit more than 15 years prior) or individuals younger than 55 or older than 74 years at the beginning of screening.

We extrapolated the findings of the NLST and compared various screening programs if adopted in the US population. Five modeling groups used independent approaches to combine multiple sources of data to simulate the underlying natural history of lung cancer and to estimate the benefit of alternative screening programs. In a single cohort of people born in 1950, each model estimated the benefits from 576 screening programs that varied eligibility criteria and frequency of screens, and two reference scenarios. We sought to rank programs according to a measure of efficiency, to reduce the number of programs that would require closer evaluation. The 1950 birth cohort was selected because they reach age 63 (about mid-range of participants in the NLST) in 2013. When independent models reach consensus on the characteristics of efficient screening programs, as reported here, the results can better inform screening guidelines. As in prior comparative modeling studies of important public health questions [8], [9] independent modeling groups collaborated, sharing inputs and standardizing analyses to remove uncertainty due to incongruent modeled populations, endpoints and metrics.

## Methods

### Models

The microsimulation models used were developed independently by investigators at five institutions funded by the National Cancer Institute's Cancer Intervention and Surveillance Modeling Network (CISNET, www.cisnet.cancer.gov) consortium through a peer-reviewed, cooperative award (2010–2015) from the National Institutes of Health: Erasmus MC in the Netherlands (Model E), Fred Hutchinson Cancer Research Center (Model F), Massachusetts General Hospital (Model M), Stanford University (Model S) and the University of Michigan (Model U). Additional investigators (see also Acknowledgments) collaborated to develop common inputs and standardize analyses. The analyses and results described in this report were part of a project to inform recommendations for lung cancer screening issued by the US Preventive Services Task Force [10].

Each of the five models simulated the underlying natural history of lung cancer, including dose-response modules that relate an individual's detailed, dynamic cigarette smoking history to lung cancer risk (by histology and sex), and estimated (as an output) the effect of early detection with CT screening on lung cancer survival (Table 1, Part A in File S1, and Table S1 in File S1). Algorithms for following up a positive screening test (defined in our analysis as suspicious for lung cancer) were simulated with varying detail (Table 1). Prior to this analysis, all models were populated with de-identified trial participant histories and adjusted to match the trial design (e.g., numbers of screens and screening modality). All models were calibrated to reproduce multiple endpoints consistent with NLST and the Prostate, Lung, Colorectal and Ovarian (PLCO) [11] cancer screening trial [12]. Because the models simulate the natural history of disease, they can predict outcomes in years after the last year of observed follow up and in what-if scenarios with hypothetical screening programs and participants.

**Table 1:** Comparison of features across five independent models.

	Erasmus MC	Fred Hutchinson Cancer Research Center	University of Michigan	Massachusetts General Hospital	Stanford University
Model Features	Model E	Model F	Model U	Model M	Model S
Central dose-response model	Two-stage clonal expansion (TSCE) [40]	Longitudinal multistage observation	Multistage clonal expansion	Probabilistic [41]	TSCE [40] with modifications
Diagnostic follow-up algorithm	Implicit. Stochastic chance (separately for patients with lung cancer diagnoses versus false positives) of receipt of a set number of follow-up exams, based on the observed frequency of exams per positive exam in the NLST CT arm.	Implicit (see model E).	Implicit (see model E).	Explicit. Detailed algorithms based on size thresholds and risk factors. Simulated less-aggressive algorithms than the Fleischner guidelines [42] to approximate the observed frequency in the NLST, which did not specify an algorithm.	Explicit (see Model M).
Screening effectiveness mechanism	Cure model. Screen-detected cases experience a reduced risk of dying from lung cancer (compared to the stage-specific survival had the same tumor been diagnosed clinically). The improved prognosis is represented as a cure fraction (specific to stage, estimated via calibration to screening trial results). If curative treatment fails, the patient survives as long as if the tumor had been diagnosed clinically, corrected for lead-time.	Combination cure model and stage shift Model F assumes that screen-detected cancers were treated according to clinical practice guidelines with estimated cure rates that depend on both tumor stage and histology.	Stage shift model, with adjustments for age. Time to death from lung cancer detection is based on survival models that define cure by histology, stage, gender, and age at diagnosis with better outcomes associated with younger age at detection. Screening can lead to improved survival due to detection at earlier stages.	Cure model with possibility of recurrence. Patients with early-stage non-small cell lung cancer undergo resection (lobectomy, consistent with consensus practice guidelines) which removes the primary cancer. For patients with neither undetected distant (lethal) metastases nor undetected primary lung cancers in another lobe of the lung, resection is curative for lung cancer.	Cure model. The probability of lethal metastases is estimated as a function of tumor size, histology and sex. With screening, patients are more likely to be detected at early stages and before the onset of lethal metastases, and cured following standard of care; patients are not cured if detected in early stages but after the onset of lethal metastases or in advanced stages.
Operative mortality and operative candidacy	Neither varied with age.	Neither varied with age.	Neither varied with age.	Neither varied with age in comparative analysis. In second analysis, simulated decreased rates of operative candidacy for older persons, and excluded from screening anyone who was not an operative candidate. Operative mortality (applied to operative candidates with early stage cancer) was constant.	Neither varied with age.

Supplementary Model Descriptions and Table S1 in File S1 provide additional details, including data used to develop and verify models.

### Common Model Inputs

Publicly available data were used for this analysis. All models simulated US men and women (all races) born in 1950. Detailed smoking histories (including non-smokers) and non-lung-cancer mortality risks were created as described below and in Part C in File S1, and Figures S1 and S2 in File S1, and used by all models as common inputs. Smoking histories and quit rates that were previously estimated through 2000 [13] were updated to calendar year 2009 for this analysis [14] and years past 2009 were projected; similarly, tables of non-lung-cancer mortality rates specific to smoking history (i.e., categories of current smokers had increased risks relative to never smokers, with former smoker mortality interpolated as a function of years since quitting) [15]) were updated to 2009 and projected past 2009. (The proportion of the 1950 cohort that had accumulated the specified number of pack-years by a given age is shown in Figure S4 in File S1.) In the NLST and the PLCO trial, individuals had substantially lower non-lung cancer mortality than the general population even after adjusting for their smoking status. Our use of US population other-cause mortality rates rather than the lower rates observed in the NLST or PLCO was based on an assumption that the “healthy volunteer” effect in the trials would not persist if screening for lung cancer disseminated widely.

### Standardized analyses

Each model was used to simulate men and women who were born in 1950 from age 45 (calendar year 1995) to death or age 90, under 576 programs and 2 reference scenarios (a no screening scenario and a scenario with a maximum of 3 screens; Table 2). Screening programs varied according to five criteria: age to start screening (45, 50, 55, 60); age to stop screening (75, 80, 85); screen frequency (every 1, 2, or 3 years); minimum number of pack-years of cigarette exposure (10, 20, 30, 40); and (for former smokers) maximum years since quitting (10, 15, 20, 25). We refer to programs using shorthand for Periodicity (A, annual, B, biennial, or T, triennial), Start Age - Stop Age - Minimum Pack-Years - Maximum Years Since Quit. For example A55-75-30-15 represents starting screening at age 55 years and ending screening at age 75, for individuals with a minimum smoking history of 30 pack-years, and a maximum years since quitting of 15 years. This program, which we refer to as ‘NLST eligibility’ is similar to the NLST design except that screening was not limited to 3 screenings (a maximum of 21 screens are possible from ages 55 to 75).

**Table 2:** Screening programs evaluated.

Program Characteristic	Values	# of Combinations
Frequency of screening	Annual, every 2 years, every 3 years	3
Age to begin screening	45, 50, 55, 60	4
Age to end screening	75, 80, 85	3
Minimum PY for screening	10, 20, 30, 40	4
Maximum YSQ for screening	10, 15, 20, 25	4
*Total (including 2 reference programs)*		578

PY, pack-years; YSQ, years since quitting. Reference programs: no screening and an approximation of the National Lung Screening Trial design (at age 62, 3 annual screens for smokers with > = 30 PY, and < = 15 YSQ).

All screening programs simulated U.S. cohorts born in 1950. For individuals meeting the pack-year and (for former smokers) years since quitting cutoffs, the first screen occurs at the beginning age and last screen occurs at the ending age. Programs are labeled as follows: Frequency (Annual, Biennial, Triennial) Age Start-Age Stop-minimum PY- maximum YSQ. As an example, **B55-85-20-15** corresponds to biennial screening starting at age 55, ending at age 85, subject to a minimum pack-year history of 20 and a maximum years since quitting (for former smokers) of 15.

As individuals age, their accumulated pack-years or years since quitting may change. In this analysis, the models assessed eligibility annually; to be screened at a specific age within the qualifying age range, an individual also had to meet *both* the pack-years and the years-since-quitting criteria. Thus lighter smokers may not begin screening at the start age and former smokers may cease screening prior to the stop age.

All simulations were performed assuming idealized, perfect screening adherence for eligible individuals and smoking cessation was assumed to be unaffected by screening results.

For the biennial and triennial programs, the frequency of screening exams was changed while retaining each model's natural history parameters, which simulate the underlying progression of disease.

Model M generated a second set of results that added operative candidacy (i.e. healthy enough for curative surgery) as an eligibility criteria for screening and reduced rates of operative candidacy in older patients (Part A in File S1) [16].

### Outcome Metrics

For each program, each model generated counts of screening exams and lung cancer deaths avoided relative to no screening, separately for males and females. All events are ‘per person in the population’ rather than ‘per person screened’ because programs defining eligibility based on smoking history may screen *similar proportions* of the population but screen *dissimilar people*, even for identical starting and stopping ages. Counts of screening exams excluded follow-up and incidental CT exams. Counts of deaths avoided per screening scenario were expressed as the proportion of the (within-model) maximum possible deaths avoided from any of the screening programs evaluated.

In this analysis, we sought to formally represent the tradeoffs between maximizing the benefits (here, lung cancer deaths avoided) accruing to a specific screening program while simultaneously minimizing the harms (here, the numbers of screening exams required to avoid the lung cancer deaths). One way to compare alternative programs that represent different tradeoffs is to generate an “efficiency frontier”. Each model generated efficiency frontiers for each sex that connected the screening programs that prevented the most deaths for each possible value of the number of CT screens. (Note that our definition of efficiency is not equivalent to identifying the lowest ratio of screens per death avoided. As screening intensity increases, the number of screens per death avoided will increase, but among programs with similar numbers of screens, some [the most efficient] will prevent more deaths.) For each model's results, we generated a rank score (decile of distance [17] from the model's frontier) for each program not on the frontier (Part B in File S1). Programs on or closest to the frontier (first three deciles) as predicted by at least 3 models were identified for males and females separately. Programs that were in both male and female lists were defined as consensus programs.

For each consensus program, we combined counts per 100,000 persons from males and females and calculated the mean predicted counts of lung cancer cases, lung cancer deaths, life years, and screening CT exams performed. We calculated the percent of the cohort receiving at least one screening exam and the number of persons ever screened per lung cancer death avoided (number needed to screen, NNS).

A secondary set of consensus programs for which the benefit (i.e., the y axis) was measured as life years saved (with the x axis remaining counts of CT screens) was also identified, using the identical steps as above.

## Results

**Figure 1:**
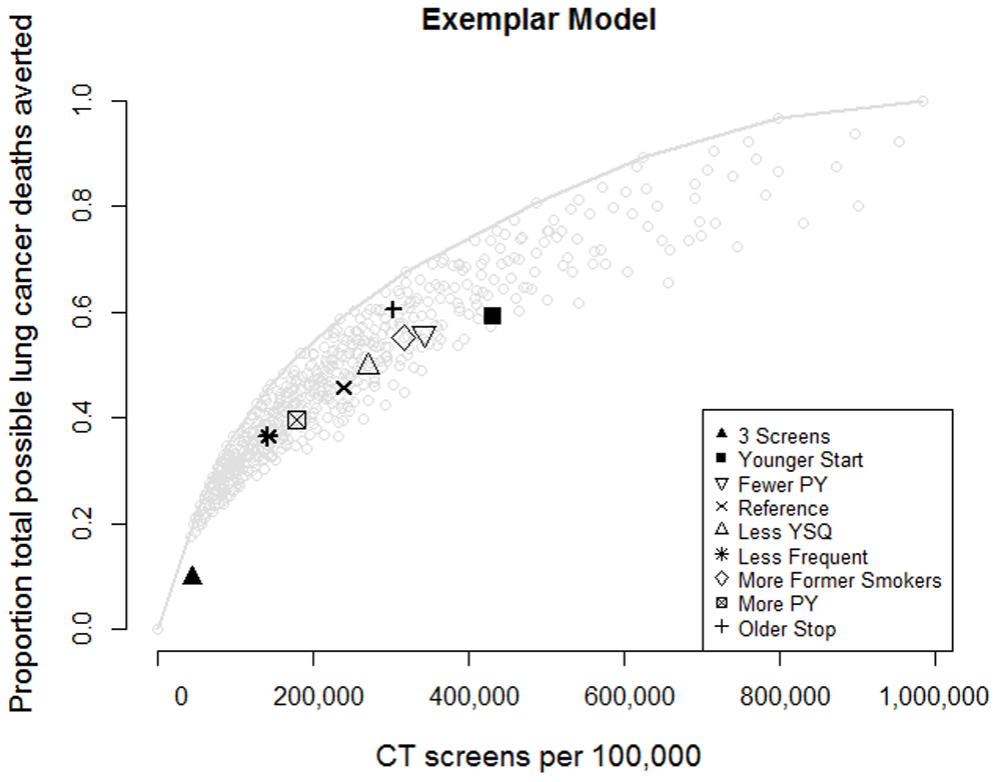
Systematic variation of reference screening program A55-75-30-15. Vertical axis normalized so that 1.0 represents within-model prediction of lung cancer deaths avoided with most intensive screening program (A45-85-10-25); values not directly interpretable as a hazard ratio. Compared to annual screening of individuals aged 55 to 74 with at least 30 pack-years of cigarette smoking and who quit with in the last 15 years (reference, x) a program of continuing annual screening to eligible individuals up to age 85 (+) was closer to the efficiency frontier. Results from one model shown; see Figure S7 in File S1 for results from all five models.

Using eligibility criteria like those in NLST, neither 3 annual screens (A62-64-30-15) nor 21 annual screens (A55-75-30-15) appears on the frontier for any model (Figure 1 and Figure S7 in File S1). There was variability among the models with respect to the effects of the smoking criteria on distance from the frontier, but consensus was clear regarding age: compared with A55-75-30-15, all models placed A55-85-30-15 closer to (or on) the frontier, indicating that continuing screening to older ages was more efficient than stopping at age 75. Conversely, initiating screening at younger ages (A45-75-30-15) was farther from the frontier (less efficient). Less-frequent (B55-75-30-15) screens provided fewer benefits, as did increasing the pack-year minimum (A55-75-40-15). The most intensive annual program (A45-85-10-25) was the upper right of the frontier for all models.

**Figure 2:**
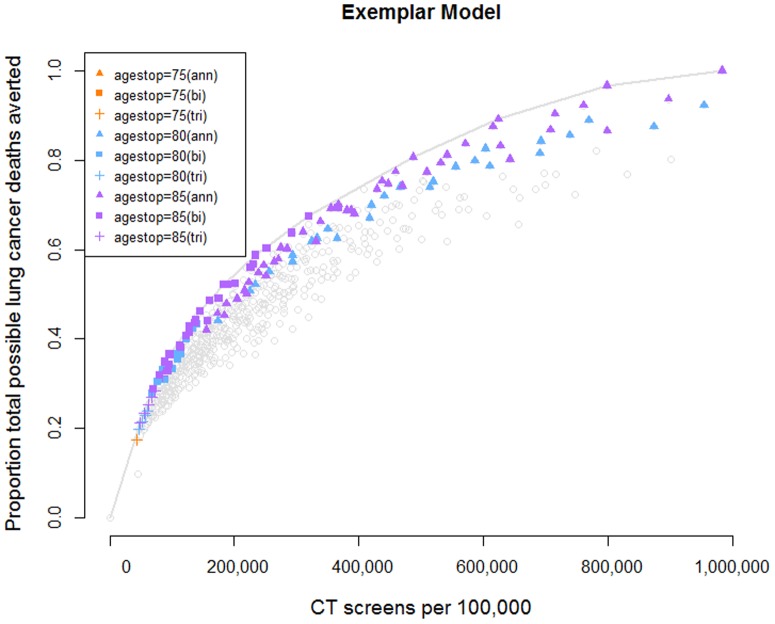
Exemplar model showing consensus programs. Vertical axis normalized as in Figure 1. Consensus programs were the 120 (out of 576 evaluated, see Table 2) that five models ranked as most efficient. Only a single consenus strategy (the single orange +) had a stop age of 75. The remaining consensus strategies continued screening of individuals meeting the smoking eligibility criteria to ages 80 (aqua) or 85 (purple). Annual screening (triangles) provided greater benefits (i.e., averted more lung cancer deaths) than triennial (+) or biennial (squares). Results from one model shown; see Figure S8 in File S1 for results from all five models.

**Figure 3:**
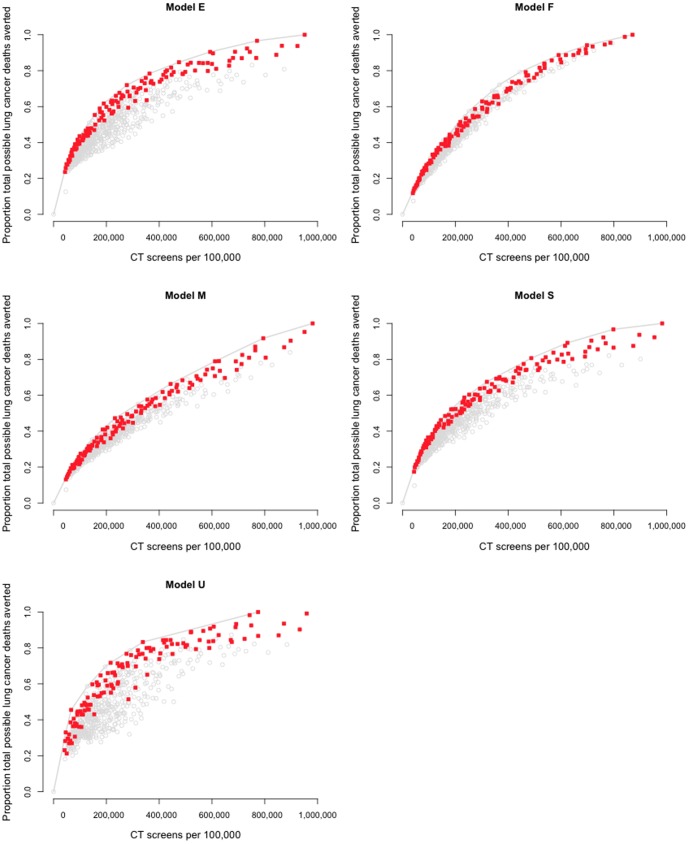
Normalized plots from all models showing consensus programs. Shown are efficiency frontiers for all 5 models, with the 120 consensus programs marked. All vertical axes are normalized to within-model predictions, as in Figures 1 and 2.

We identified 120 consensus programs. Of these, 119 had a stopping age of 80 or 85 (Figure 2, Table S2 in File S1, and Figure S8 in File S1). Across the 120 consensus programs, the average start age (54.8 y) and the average minimum pack-years (27.1) were close to the NLST criteria but the average maximum years since quit was higher (19.9 y). For all models (Figure 3), the 120 consensus programs are close to the model's own frontier.

Results from a selected subset of 41 (every third, sorted by percent ever screened) consensus programs are provided in Table 3 (mean and SD of results from the five models). Between 11% and 40% of the cohort was screened, requiring between 43,000 to over 920,000 CT screens per 100,000 persons (Table 3). The models predicted an average of 3,719 lung cancer deaths per 100,000 in the no screening scenario (SD 820.43; Figure S6 in File S1). Per 100,000 persons, the 41 consensus programs would avoid between 153 and 846 lung cancer deaths and save between 1,883 and 9,851 years of life, relative to no screening, and the mean predicted NNS varied from 34.5 to 94.2.

**Table 3:** Mean (SD) predicted benefits from 5 models for 41 selected (of 120) consensus programs (both sexes combined).

Program characteristics: FreqStart-Stop-PY-YSQ	% cohort ever screened∧ (mean)	% cohort ever screened∧ (SD)	Number of CT screens (mean)	Number of CT screens (SD)	Lung cancer deaths avoided** (mean)	Lung cancer deaths avoided** (SD)	NNS (mean)	NNS (SD)	Life-years saved** (mean)	Life-years saved** (SD)
T60-75-40-10	11.1	1.0	42,893	2,757	153	72	94	64	1896	1093
T60-80-40-10	11.2	1.0	45,685	3,223	173	78	85	60	1883	1201
B60-85-40-10	11.3	1.1	69,662	4,466	256	115	59	44	2771	1639
T60-85-40-15	12.0	1.2	55,316	3,573	201	93	77	52	2085	1426
T60-80-40-20	12.6	1.0	56,712	3,502	197	88	81	52	2138	1344
B60-85-40-20	12.7	1.0	88,781	4,802	288	138	57	37	2943	1957
T60-80-40-25	12.9	0.9	60,570	3,483	202	92	80	47	2299	1352
T60-85-40-25	13.0	0.9	66,333	3,578	225	106	73	44	2344	1559
A60-85-40-25	13.0	0.9	185,451	8,027	449	219	38	25	4394	2859
A55-85-40-15	13.7	0.8	200,575	10,864	445	223	41	29	4740	2844
T55-85-40-25	13.9	0.9	83,043	4,633	252	120	70	44	2767	1702
A55-85-40-20	14.0	0.9	220,505	10,542	485	237	38	26	4958	3029
B50-80-40-25	14.5	0.6	137,944	6,221	358	167	51	32	4012	2216
B50-85-40-25	14.6	0.7	143,621	6,835	376	178	49	30	4090	2377
A50-85-40-25	14.6	0.7	281,218	11,061	542	261	35	22	5955	3161
A60-85-30-10	15.6	1.0	180,599	7,772	412	200	50	34	4212	2603
A60-85-30-15	16.9	1.1	213,400	8,568	457	232	49	32	4666	2964
B60-85-30-20	17.9	1.2	127,046	4,888	358	166	64	41	3591	2304
A60-85-20-10	18.3	1.0	214,153	7,742	452	218	53	35	4613	2839
A55-80-30-15	19.3	1.0	286,813	11,098	521	268	49	31	5603	3278
A55-85-30-20	20.2	0.8	331,990	11,705	593	305	44	27	6237	3642
A55-85-30-25	20.4	0.9	361,001	11,107	628	323	42	25	6469	3822
A50-85-30-15	21.2	0.7	382,439	15,625	608	316	45	27	6998	3596
A50-85-30-20	21.4	0.8	419,782	15,070	653	336	42	25	7244	3781
A45-85-30-25	22.0	0.7	520,793	18,498	707	362	39	22	7775	3959
B60-85-20-20	23.2	1.0	158,397	4,474	399	185	73	44	4070	2508
A60-85-20-25	24.8	1.0	348,894	6,919	624	314	51	30	6120	3857
A55-80-20-20	26.6	0.9	410,565	10,425	631	342	55	32	6928	3892
B55-85-20-25	27.4	1.1	247,058	6,305	501	256	69	39	5256	3153
A50-85-20-15	27.9	0.9	496,010	15,834	685	378	53	30	7688	4118
A60-85-10-20	28.0	2.0	370,825	19,139	605	296	59	34	6108	3671
A50-85-20-20	28.7	1.0	557,513	15,580	737	411	50	28	8028	4450
A50-85-20-25	29.0	0.9	610,443	14,822	787	427	47	25	8746	4512
A45-80-20-25	29.9	1.1	721,956	19,536	780	453	49	25	9206	4531
A55-85-10-15	29.9	2.3	448,193	26,722	651	332	59	34	6876	3909
A60-85-10-25	31.1	2.1	427,669	21,334	660	322	59	32	6474	3951
A50-80-10-15	34.6	2.3	583,756	35,681	700	388	63	34	8036	4143
A55-85-10-25	36.0	2.0	590,101	31,172	768	397	59	31	8109	4454
A50-85-10-20	37.5	2.0	685,484	39,445	795	422	59	31	8772	4509
A50-85-10-25	38.9	1.9	767,313	40,320	851	443	57	28	9151	4735
A45-80-10-25	40.3	1.9	920,505	45,739	846	479	60	29	9851	4737
*Average CV*	*0.06*		*0.04*		*0.50*		*0.61*		*0.58*	

Percentage of cohort screened, numbers of CT screens, lung cancer deaths avoided, and life years saved are all normalized to cumulative counts per 100,000 people in the cohort at age 45 (including non-smokers and persons not screened), followed to age 90. See Table S2 in File S1 for complete list of 120 consensus programs identified from the 576 programs evaluated.

*Frequency, A = annual, B = biennial (every 2 years), T = triennial (every 3 years); Start Age, Stop Age, PY = minimum pack-year, YSQ = maximum years since quit.*

*NNS, Number (people) needed to screen (ever) to prevent one lung cancer death.*

∧ *Percent of cohort that received at least one screen; eligible individuals varied across programs.*

** Numbers of lung cancer deaths avoided and life years saved were first calculated per model, comparing each model to its own results for lung cancer deaths in the no-screening arm. Shown are averages across models. The average (across models) number of lung cancer deaths in the no screening scenario was 3719 (SD 820).

*Average Coefficient of Variation (CV) calculated as the average of (SD/mean) for each program in the table. Lower values indicate less dispersion of estimates from the models for that endpoint, across the selected consensus programs.*

Based on results from one model (M), reducing the proportions of older individuals screened (due to ineligibility for surgical resection) resulted in fewer CT screens and fewer lung cancer deaths avoided (13.3% and 14.8%, respectively, across the consensus programs), but programs that extended screening to ages 80 and 85 remained on the efficiency frontier (Figure S9 in File S1).

When the benefit of screening was measured as life years saved rather than lung cancer deaths avoided, the second set of consensus efficient programs had younger average start and stop ages (49.5 y and 80.9 y, respectively) but similar average minimum pack-years and maximum years since quit (Table S3 in File S1).

## Discussion

Five independent models ranked 576 lung cancer screening programs by weighing one metric of their potential benefits (lung cancer deaths avoided) against one measure of harms or resource use (counts of CT screening exams) in the US cohort born in 1950. The models had been previously calibrated to multiple endpoints in NLST,^12^ but heterogeneity in the underlying model structures and assumptions yielded heterogeneous predictions for absolute numbers of lung cancer deaths avoided when extrapolating beyond the trial data. A key finding of our analysis was that despite differences in absolute benefits across the models, the ranking of programs was consistent; while accounting for the heterogeneity in model predictions, we were able to identify a set of consensus efficient programs. Annual screening with eligibility based on NLST criteria (beginning at age 55, continuing to age 75 for current and former smokers with a minimum of 30 pack-years and less than 15 years since quitting) was not among the programs on the efficient frontier of any of the five models. Results from all models showed that programs that extended the screening age beyond 75 prevented more lung cancer deaths for relatively few additional screens. Note that in our modeling, the stopping age for a program was the last screen for any individuals who still met the smoking cutoffs, and *not* the last year to be invited to begin a screening program. In the NLST which had an upper eligibility age of 74 years, individuals were as old as (77 or, rarely, 78) at the third screen. Our finding that programs that screened eligible individuals past age 75 years were efficient was unchanged when more older patients were ineligible for screening due to comorbidities that categorized them as non-operative candidates (based on results from one model) or when life years saved was substituted for the measure of benefit. While in other cancers (e.g. breast and colorectal) screening is not generally recommended beyond age 75 and not generally recommended every year, in lung cancer annual screening to older ages can be beneficial because: (1) the age-specific incidence curve for lung cancer is quite steep, and (2) the high lethality of the disease makes early detection worthwhile, even among individuals with a somewhat modest life expectancy. It is also important to note that had we defined life years saved (instead of lung cancer deaths avoided) as the measure of benefit, one could logically predict that strategies with younger stopping ages would be more likely to emerge as ‘consensus efficient’.

Our predicted NNS for A55-80-30-15 varied across models, ranging from 19.8 (Model F) to 100.5 (Model M), but all were below published estimates of NNS for only 3 screens of (256) [18] and closer to published NNS for mammography (95) or FOBT (roughly 130) for healthy 50 year-olds [19].

For consensus programs with screening until age 80, between 11% (for the least frequent programs with strictest eligibility, e.g., T60-75-40-10) and 40% (for the annual programs with more inclusive eligibility, e.g., A45-80-10-25) of the cohort born in 1950 would be screened at least once after age 45. Although not directly comparable to earlier estimates that 6% (8.7 million people) of US adults over 40 would meet the NLST eligibility cutoffs for lung cancer screening each year [20], [21], our estimate of 11% of individuals seems reasonable.

We identified a set of consensus efficient programs rather than a single optimal strategy, because the efficiency frontiers did not identify a consensus inflexion point at which additional screens provided diminishing benefits. The least intensive programs at the lower left of the frontiers (Figure 2) may be less attractive, however, since annual screening consistently prevented more lung cancer deaths than did triennial or biennial programs. The most-intensive screening programs, on the other hand, will lead to more accumulated harms (radiation exposure from additional imaging examinations, overdiagnosis, invasive biopsies) and costs.

Screening programs cannot be evaluated in isolation from the follow-up algorithm. In the NLST, an average of 24% of individuals in a given round of screening (CT arm) had results requiring some follow-up, but the trial did not specify a follow-up regimen, leaving open the question of the optimal regimen for individuals with positive screens, most of whom are healthy [4], [22]. In models (E, F, U) that used implicit follow-up algorithms based on the experience of participants in the NLST, extrapolating the rate of follow-up to less frequent screening programs was dependent on the assumption that the rates of follow up exams and early detection of lung cancers (defined in the NLST and models E, F, and U as ‘screen-detected’ even if first seen on a follow-up exam) would not change. In the models (M, S) that explicitly modeled follow-up programs based on size, follow-up exams could change the timing of detection of a lung cancer, but the assumptions used here for frequency of follow-up imaging may not be representative of eventual practice patterns.

Several limitations of our analysis are important to note. The models do not simulate non-lung cancer incidental findings (e.g., coronary artery calcification, AAA, or other malignancies), so our results do not include potential benefits (or harms) due to their detection and treatment. There are few data to predict adherence patterns for lung cancer screening [20], [23], and many possibilities to model. We conducted an idealized analysis with the goal of informing guidelines and did not consider that individuals will self-select for participation in screening based on their comorbidities, specific smoking history, or family history, as observed in screening trials [24], [25]. It will be important to monitor how lung cancer screening is implemented in community settings (including recruitment, participation, positive screen evaluations, diagnosis, referral for treatment), and modeling can suggest the most important leverage points to optimize the process. Definitive evidence on the relationship between smoking cessation and NLST screening results was not available in time for our analyses. Based on limited data with non-standardized definitions of ‘quit’ [26]–[29] and the PLCO Trial, which found no correlation between CXR screening result and smoking behavior [30], we assumed screening did not affect background smoking patterns.

Efficient screening programs might differ in populations with different smoking patterns or other-cause mortality risks than the cohort we simulated. To simplify the comparison of hundreds of programs, we performed our analyses in a single birth cohort and did not estimate total lung cancer deaths avoided in the US [31]. Our requirement that individuals meet all eligibility criteria (including years since quitting) was transparent and is a step towards risk-based screening criteria (our models account for decreasing risks of death from lung cancer and other causes after quitting), but may not reflect guidelines, which typically define eligibility to begin screening. Future analyses to examine programs that define eligibility based on risk models will require that the models and population input files include additional characteristics (e.g., BMI, education) that go beyond age and smoking exposure [32]–[36]. We did not incorporate increases in operative mortality rates by age, or special clinical considerations individual to a particular patient.

Although the rankings of programs were consistent across models, uncertainty in absolute numbers of lung cancer deaths avoided (and life years saved) remained, due to variation in the underlying assumptions regarding unobserved disease processes [37]. Underlying the differences across models in predicted absolute benefits is a variation in the predicted future number of lung cancer cases in the absence of screening (Figure S5 in File S1). Essentially, our consortium of 5 models served as a sensitivity analysis on model structure and demonstrated that even when model heterogeneity was specifically taken into account, the models identified similar efficient programs (i.e., the consensus set).

Our results highlight tradeoffs between preventing greater numbers of lung cancer deaths and the additional screening exams required. Guidelines for screening also consider tradeoffs in gains in life expectancy and important harms, including invasive biopsies for benign disease, overdiagnosis, and lung cancers related to radiation from diagnostic imaging examinations [10]. Difficulties with estimating population effects of screening include the potential for concurrent smoking cessation programs to augment the benefits from screening, and the heterogeneity of the radiation dose attributable to a given CT exam, which could vary as much as 10-fold depending on the size of the patient, the generation of scanner, and the protocol in use at the clinical setting [38]. All smokers, whether undergoing screening or not, should receive cessation assistance and be encouraged to quit [39].

## Supporting Information

File S1 **Supporting figures and tables.** **Figure S1, Prevalence of smoking by age in 1950 birth cohort.** Summary of shared input data (used by all 5 models) on smoking patterns for the US cohort born in 1950. Prevalence shown is estimated in the absence of lung cancer mortality. Version 1.0 of the Smoking History Generator (SHG) refers to published data through 2000 (Anderson, et al.), and version 1.5 supplies the 1950 birth cohort used for this analysis with data through 2009 and projections past 2009. **Figure S2, Other-cause mortality, by smoking quintile, in 1950 birth cohort.** These curves show the other-cause (non-lung cancer) mortality for never smokers and for current smokers by smoking quintile (Q, of cigarettes per day) for the male birth cohort of 1950, out to age 99. Former smokers are intermediate to current and never smokers. There is a similar plot for females. These were shared inputs used by all the models. Note that the rates of non-lung cancer mortality represent the US population, not trial (NLST or PLCO) participants. **Figure S3, Prevalence of smoking by age in 1950 birth cohort.** Output from one model showing smoking prevalence by age (calendar year), in a no screening scenario. Proportions of current/former/never smokers are in the presence of lung cancer mortality as well as all-cause mortality. **Figure S4, Prevalence of smoking by age and pack-years in 1950 birth cohort.** Output from one model showing smoking prevalence by category of pack-year and age. The proportion of the cohort by age that has accumulated the specified number of pack-years in the presence of lung cancer mortality and other-cause mortality. **Figure S5, Incidence, no screening scenario, output from all models.** For predictions past observed SEER data (over age 60) there are no observed data, but we used an age-period-cohort model to project past observed years (‘Projected’ red double line in plots below), which shows that the models are most divergent after age 85, when SEER data become most sparse. We cannot strictly compare incidence to that in prior birth cohorts since smoking patterns are dissimilar, and incidence varies by cohort. **Figure S6, Mortality, no screening scenario, output from all models.** The vertical line at age 90 indicates age at which all event counts (screens, deaths and deaths averted, and life years gained) were truncated for the analyses reported here. Although the models ranked programs similarly, there was variability in the total numbers of predicted lung cancer cases, deaths, and therefore lung cancer deaths prevented. The differences in rates in the no screening scenario in large part explains the predicted differences between models. The four models (E, F, S, and U) which use two-stage or multi-stage clonal expansion models have more similarly shaped curves than the fifth model (M), which does not use a clonal expansion component (see Table S1 in File S1). **Figure S7, Results from all models analogous to** **Figure 1** **in article. Figure S8, Results from all models analogous to** **Figure 2** **in article. Figure S9, Secondary results with reduced operative candidacy with age.** The dashed line denotes the efficiency frontier in the main analysis. **Table S1, Additional Detail on Models. Table S2, Complete List of 120 Consensus Efficient Scenarios. Table S3, Comparison of Consensus Efficient Scenarios Identified Using Life-years Saved or Lung Cancer Deaths Avoided as Measure of Benefit.**
(DOCX)
