# Supplementary material for: Comparing Benefits from Many Possible Computed Tomography Lung Cancer Screening Programs: Extrapolating from the National Lung Screening Trial Using Comparative Modeling
Source: PLoS One. 2014 Jun 30;9(6):e99978. doi: 10.1371/journal.pone.0099978 (PMC4076275; doi:10.1371/journal.pone.0099978)
Supplement: File S1 — Supporting figures and tables. Figure S1, Prevalence of smoking by age in 1950 birth cohort. Summary of shared input data (used by all 5 models) on smoking patterns for the US cohort born in 1950. Prevalence shown is estimated in the absence of lung cancer mortality. Version 1.0 of the Smoking History Generator (SHG) refers to published data through 2000 (Anderson, et al.), and version 1.5 supplies the 1950 birth cohort used for this analysis with data through 2009 and projections past 2009. Figure S2, Other-cause mortality, by smoking quintile, in 1950 birth cohort. These curves show the other-cause (non-lung cancer) mortality for never smokers and for current smokers by smoking quintile (Q, of cigarettes per day) for the male birth cohort of 1950, out to age 99. Former smokers are intermediate to current and never smokers. There is a similar plot for females. These were shared inputs used by all the models. Note that the rates of non-lung cancer mortality represent the US population, not trial (NLST or PLCO) participants. Figure S3, Prevalence of smoking by age in 1950 birth cohort. Output from one model showing smoking prevalence by age (calendar year), in a no screening scenario. Proportions of current/former/never smokers are in the presence of lung cancer mortality as well as all-cause mortality. Figure S4, Prevalence of smoking by age and pack-years in 1950 birth cohort. Output from one model showing smoking prevalence by category of pack-year and age. The proportion of the cohort by age that has accumulated the specified number of pack-years in the presence of lung cancer mortality and other-cause mortality. Figure S5, Incidence, no screening scenario, output from all models. For predictions past observed SEER data (over age 60) there are no observed data, but we used an age-period-cohort model to project past observed years (‘Projected’ red double line in plots below), which shows that the models are most divergent after age 85, when SEER data become most s [file pone.0099978.s001.docx]

**File S1**

**for**

***Benefits from Lung Cancer Screening: Extrapolating from the NLST to other designs and participants***

***McMahon, et al.***

Part A. Supplementary Model Descriptions

Table S1

Part B. Supplementary Analysis Methods

Part C. Supplementary Input Data (smoking histories and other-cause mortality)

Figure S1 – prevalence of smoking by age in 1950 birth cohort - input

Figure S2 – other-cause mortality, by smoking quintile, in 1950 birth cohort

Part D. Supplementary Results

Figure S3 – Prevalence of smoking by age in 1950 birth cohort – one model output

Figure S4 – Prevalence of smoking by age and pack-years in 1950 birth cohort – one model output

Figure S5 – Incidence, no screening scenario – output from all models

Figure S6 – Mortality, no screening scenario – output from all models

Figure S7 – Results from all models analogous to Figure 1 in article

Figure S8 – Results from all models analogous to Figure 2 in article

Figure S9 – Secondary results with reduced operative candidacy with age

Table S2 – Complete list of 120 consensus efficient scenarios

Table S3 – Compare consensus efficient scenarios identified using life-years saved and lung cancer deaths avoided as measure of benefit

Part E. Supplementary References

**Part A. Supplementary Model Descriptions**

See Table S1. Earlier versions of 3 of the models were described in a monograph, both individually and in comparison.[^1-4^](#_ENREF_1) Additional details of some models are also available at [www.cisnet.cancer.gov/profiles](http://www.cisnet.cancer.gov/profiles).

Calibration to NLST and PLCO trials. The details of the methods and results are provided in a publication in press (Meza, et al., cited in manuscript).

Benign pulmonary nodules

Models M and S simulate the presence of benign pulmonary nodules. In both models, if benign nodules are detected on imaging exams, the patient’s management and outcome may change.

Model M assumes that benign nodules arise according to age, and may resolve spontaneously also as a function of age. Individuals may have up to three benign nodules (and three lung cancers, for a maximum total of six nodules at one time). The sizes of benign nodules are drawn from a lognormal distribution (mean = 0.9, variance 0.36), and the locations of these nodules are based on Mayo Clinic data.[^5^](#_ENREF_5) Recent results from the NLST support the use of benign pulmonary nodule (which cause false positives) prevalences from areas in the ‘histoplasmosis belt’ for the rest of the US.[^6^](#_ENREF_6)

Model S assumes that benign nodules arise as a function of age.

Operative mortality and operative candidacy

For all models in the base case, operative mortality was assumed constant by age. Model M assumed a 1% operative rate. Other models do not explicitly model surgical mortality. In the secondary analysis in which Model M varied the probability of operative candidacy by age, we used the following values, approximated from a figure in Mery, et al.[^7^](#_ENREF_7) from an analysis of stage I/II NSCLC patients in SEER, 1992-1997 (n=14,555): For ages younger than 65 years, 92% of stage I/II lung cancer patients were assumed to be operative candidates. For ages 65-74 years and over age 75 years, the percentages were 86% and 70%, respectively.

Follow-up

Across individuals in NLST with a positive CT-screen, the mean number of follow-up CTs was roughly 1, expressed as distributions around the mean numbers of follow-up CTs given gender and screening round (and other covariates that we are not using as of now). The mean number was not stratified by cases vs. false positives, since the number of cases was negligible vs. the number of false positives. Most of the follow-up CTs occurred within a year of the screen. For the biannual and annual extrapolation simulations, Model U scans a fraction of individuals (~ 26-27% - false positive rate in NLST) exactly a year after the screen.

Models M and S simulate an explicit follow-up algorithm based on the size of the nodule (in Model M, based on the nodule with the largest diameter, if more than one nodule exists). For example, for nodules between 4 and 6 mm, Model M would simulate high-resolution CT exams at 12 and 24 months, and nodules between 6 and 8mm would undergo follow-up exams at 9 and 24 months. In the analyses, the screening exams that would have occurred during that 24 month period would be missed.

Table S1. Additional detail on models.

|  | Erasmus | FHCRC | | Univ. of Michigan | MGH | | Stanford |
| --- | --- | --- | --- | --- | --- | --- | --- |
|  | Model E | Model F | | Model U | Model M | | Model S |
| Data sources used for model development, calibration or validation | NLST and PLCO[^8^](#_ENREF_8); SEER  2000-2008 incidence by age,  stage, histology; and NHS,  HPFS[^9^](#_ENREF_9) | NLST and PLCO[^8^](#_ENREF_8); model developed using PLuSS CT and CARET[^10^](#_ENREF_10) | | NLST and PLCO[^8^](#_ENREF_8); NHS/HPFS LC incidence;[^9^](#_ENREF_9) SEER LC survival by sex, age, histology and stageNLST and PLCO[^8^](#_ENREF_8); NHS/HPFS LC incidence;[^9^](#_ENREF_9) SEER 2000-2008 LC survival by sex, age, histology and stage | NLST and PLCO[^8^](#_ENREF_8); SEER 1990-2000 incidence by age, stage, histology; survival by stage; Mayo CT; LSS[^3^](#_ENREF_3)^,^[^5^](#_ENREF_5) | | NLST and PLCO[^8^](#_ENREF_8); NHS/HPFS- LC incidence,[^9^](#_ENREF_9) SEER 1988-2003 survival by histology |
| Representative prior uses of the model | Evaluation of tobacco control programs[^11^](#_ENREF_11) and screening programs in cancers other than lung[^12-15^](#_ENREF_12) | |  |  | Evaluation of tobacco control programs[^11^](#_ENREF_11) and screening and treatment interventions[^5^](#_ENREF_5)^,^[^16^](#_ENREF_16)^,^[^17^](#_ENREF_17) | Comparing lung and breast cancer screening[^18^](#_ENREF_18) | |
| Model simulates metastasis explicitly | No | | No | No | Yes | Yes | |

**Part B. Supplementary Analysis Methods**

From each model, separately for males and females, a .csv file containing counts of CT exams and counts of lung cancer deaths avoided (relative to no screening) was generated for each of the 576 scenarios (a total of 578 scenarios, including a no screening reference scenario and a reference scenario with 3 screens). Each model simulated at least 1 million individuals per scenario. Cumulative counts of events from age 45 to age 90 were normalized per 100,000 individuals in the cohort at age 45. For each model, we plotted CT screening exams (x-axis) and lung cancer deaths avoided, relative to a no screening reference strategy (y-axis). The y-axis was normalized to display the proportion of the maximum possible lung cancer deaths avoided, as predicted by that model, from the most-intensive strategy modeled (A45-85-10-25).

In R (version 2.15.2, RStudio version 0.97.248), the convex hull function was used to identify the efficiency frontier. Secondly, the nonparametric *nonparaeff* package in R, which uses data envelopment analysis (DEA) method to measure efficiency (productivity) was used to measure an ‘efficiency score’, a measure of distance from the efficiency frontier.[^19^](#_ENREF_19)^,^[^20^](#_ENREF_20) Data Envelopment Analysis (DEA) measures the relative efficiencies of strategies, units, or organizations with multiple inputs and multiple outputs. We used the output-oriented DEA model, which maximizes the output (deaths avoided) for each value of the input (counts of CT screens) held constant to generate efficiency scores. An efficiency score of 1 identifies scenarios on the frontier, and increasing scores (>1) indicate increasing distance from the frontier. We grouped the scores into deciles and defined each decile as an efficiency rank. Specifically, programs with ranks 1, 2, and 3 were on or near the frontier and were termed ‘optimal’ programs. We used the program’s rank from each model to compare results across models.

We identified strategies that were in at least 3 models’ acceptable region, which yields a list of consensus strategies in males and (separately) females, and identified the intersection of the male and female lists of consensus strategies to yield a list of consensus strategies applicable to both sexes. Outcomes shown are averages of five model’s estimates, for males and females combined (weighted by population distribution at age 45: female contribution =0.5188053 and male contribution =0.4811947).

**Part C. Smoking Histories and Non-Lung Cancer Mortality Risks of Simulated Cohort (born in 1950)**

The 1950 birth cohort represents the US population aged 45 in 1995, with respect to smoking patterns (including non-smokers) and other-cause (non-lung cancer) mortality. In the absence of screening, mortality rates in the cohort would correspond to US rates (available from the National Center for Health Statistics) from 1995 to 2012, or ages 45 to 62.

Estimates of smoking history parameters were derived from 33 National Health Interview Surveys (NHIS) conducted from 1965-2009 and temporal trends were used to extrapolate smoking patterns to future years, as described below.

Thirteen of the NHIS surveys (carried out from 1970-2001) included more detailed questions on age at initiation for subjects with a history of smoking and age at cessation for former smokers. This additional detail provided an approach for retrospectively constructing smoking histories for those surveyed, but because of the cross-sectional nature of these data, important details on the experience of those not surveyed were not available. Especially important were mortality differences caused by cigarette smoking, which would be expected to bias (downward) the estimated proportion of smokers in a generation as they are sampled at older ages. Correction for this bias is essential[^21^](#_ENREF_21)^,^[^22^](#_ENREF_22) and in this analysis (see also Holford, et al., in press, cited in manuscript) we have extended the approach used by Anderson, et al.[^23^](#_ENREF_23) for NHIS surveys up to 2000 to include nine additional surveys up to 2009. The approach essentially followed that used by Anderson et al., yielding estimates of (a) yearly age-specific initiation and cessation probabilities, (b) prevalence of current, former and never smokers, and (c) quintile of dose (mean cigarettes smoked per day).

Temporal trends in smoking were analyzed using age (*a*), period (*p*) and birth cohort (*c*) factors in the model. The well known identifiability problem affects interpretation of the separate effects of each temporal component in models[^24^](#_ENREF_24) (refs), but estimated outcomes resulting from model fitting are estimable functions of the model parameters and thus not affected by the problem.

***Ever smoker prevalence:*** Data from all surveys were partitioned into five year cohorts and the proportion who had ever smoked by single year of age provided estimates of prevalence among survivors. A nonlinear model for the proportion of ever smokers as a function of age gave a good description of the data, where the prevalence of ever smokers at age a for cohort *c*, $P_{\text{E}}\left(a;c\right)$ is

$$P_{\text{E}}\left(a;c\right)=\left(\beta_{0}\left(c\right)-\exp\left\{\beta_{1}\left(c\right)\left[a-30\right]\right\}\right)/100$$

where $\beta_{0}\left(c\right)$ and $\beta_{1}\left(c\right)$ are unknown parameters that were estimated for each cohort from the NHIS data. Linear interpolation for these five year cohorts yielded estimates for single year cohorts. These estimates were used for prevalence for individuals 30 or older. For the entire age span we also included initiation probability estimates, described below.

***Cessation probability:*** Using data from surveys that reported age at cessation, birth cohort was calculated from interview age and calendar year (*c=p-a)*. For each age, the number of smokers who quit divided by the number who continued to smoke provided estimates of the conditional probability of cessation given the individual was a smoker. Constrained natural splines were used as additive effects for age, period and cohort in a linear logistic model for the yearly probability of cessation, providing estimates for the 1940, 1950 (used for analyses in this report) and 1960 birth cohorts. The period effects for years 2010 and later were held constant at the value for 2009. These fitted conditional probabilities were then used to estimate the cumulative proportion of smokers in cohort *c* who had not ceased smoking by age *a*, $F_{\text{Q}}\left(a;c\right)$.

***Initiation probability:*** Surveys reporting age of initiation were used to estimate the conditional probability that a never smoker of a given age began to smoke during that year. In a manner similar to that used for cessation probabilities, a constrained additive spline for age, period and cohort was fitted to the logit of the conditional probabilities of smoking initiation. Multiplying subsequent conditional probabilities provide an estimate of the cumulative probability of smoking initiation, which is essentially the ever smoker prevalence. However, for some cohorts there is a considerable lag between age of initiation and age at survey, providing time for substantial effects of differential smoking related mortality to take hold. Alignment between this estimate and the cross sectional estimate described above was accomplished by finding the appropriate multiplicative constant that aligned the curves at the age for the cohort at the first survey, i.e., 1965. The result was an estimate of the prevalence of ever smokers for all ages for each cohort, $P_{\text{E}}\left(a;c\right)$.

***Never smoker prevalence:*** The prevalence of never smokers is the complement of the prevalence of ever smokers, $P_{\text{N}}\left(a;c\right)=1-P_{\text{E}}\left(a;c\right)$.

***Current smoker prevalence:*** Current smokers represent ever smokers who have not quit, which we estimate from the cumulative probability of cessation, i.e., $P_{\text{C}}\left(a;c\right)=P_{\text{C}}\left(a;c\right)F_{\text{Q}}\left(a;c\right)$.

***Former smoker prevalence:*** Former smokers are those that remain after removing never and current smokers, or equivalently, the ever smokers who have not quit,

$$P_{\text{F}}\left(a;c\right)=1-P_{\text{N}}\left(a;c\right)-P_{\text{C}}\left(a;c\right)=P_{\text{E}}\left(a;c\right)\left[1-F_{\text{Q}}\left(a;c\right)\right]$$

***Quintile of mean cigarette per day:*** Quintiles of reported cigarettes per day were calculated by ten year age (30-39, …, 80-89) and cohort (1935-1944, …, 1955-1964) groups. A linear regression model was fitted to these values for each quintile by cohort and gender, and the fitted line was used to derive estimates of the mean for each quintile by single year of age for the cohorts used in this report. Dose was ramped up from 0 at age at initiation until age 30, after which the person was simulated as smoking the mean observed dose for that quintile until cessation, if it occurred. Mean dose per quintile decreased with age after age 35, as observed in NHIS data.

***Other cause mortality:*** We repeated prior methods in Rosenberg, et al.[^25^](#_ENREF_25) and developed quintile-based tables of non-lung cancer mortality rates (specific to the birth cohort, stratified by sex) for current and never smokers, with former smoker mortality calculated as a function of years since quit. Mortality rates were fixed at values for age 85 until age 99.

Figure S1. Smoking prevalence by age in 1950 birth cohort; shared input.


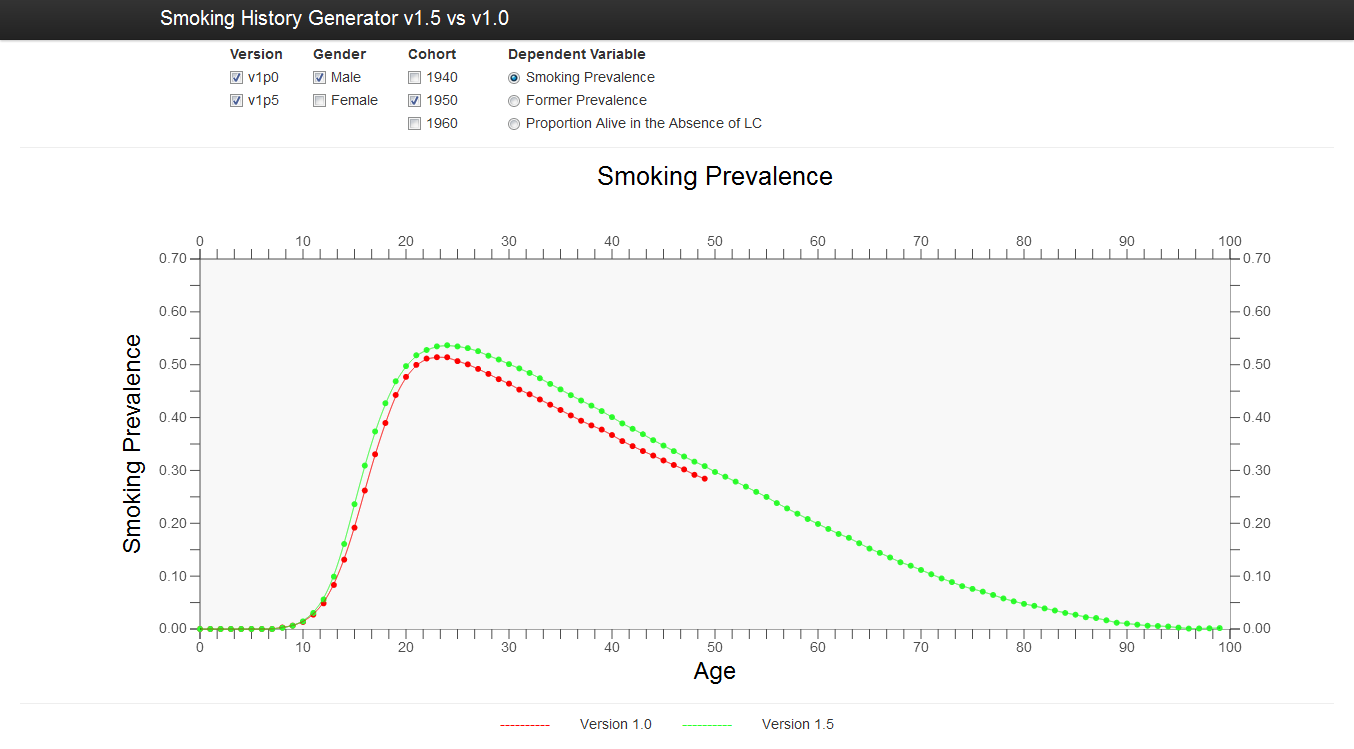


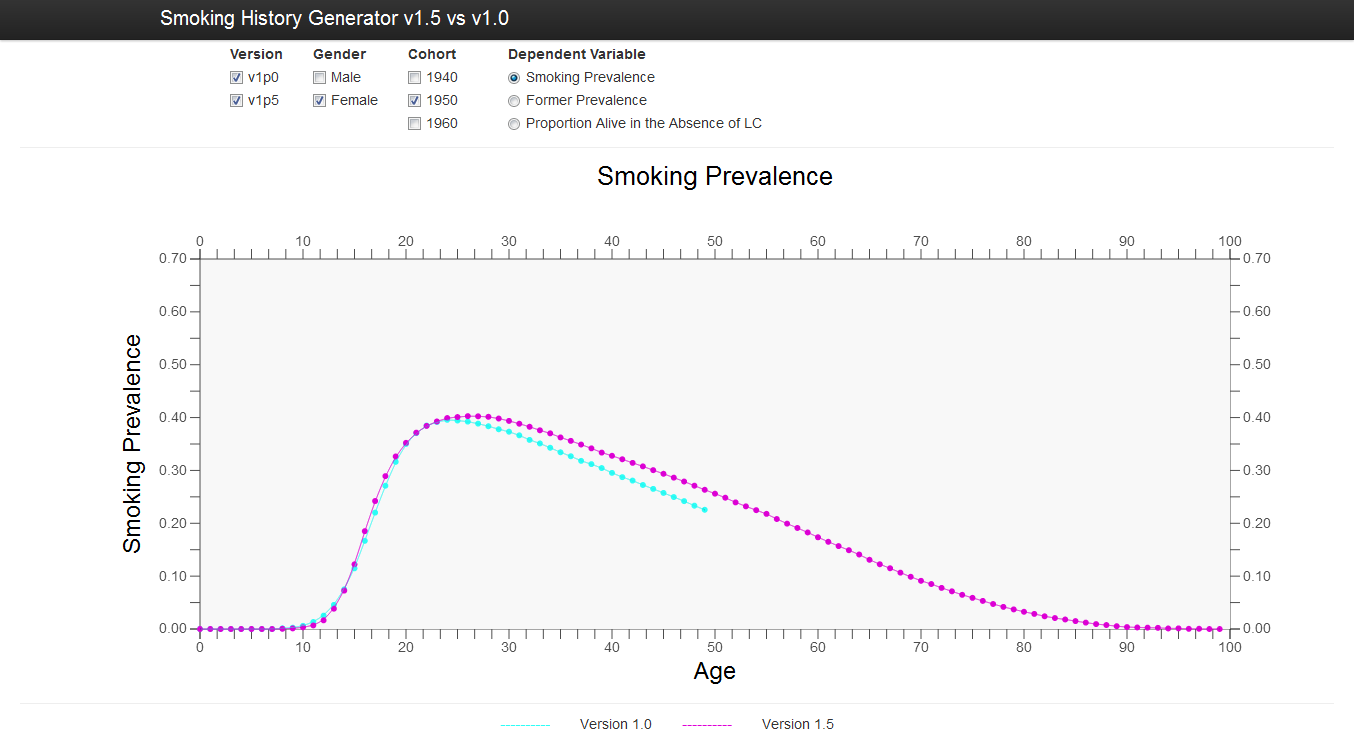


Summary of shared input data (used by all 5 models) on smoking patterns for the US cohort born in 1950. Prevalence shown is estimated in the absence of lung cancer mortality. Version 1.0 of the Smoking History Generator (SHG) refers to published data through 2000 (Anderson, et al.), and version 1.5 supplies the 1950 birth cohort used for this analysis with data through 2009 and projections past 2009.

Figure S2. Non-lung cancer mortality by age, sex, and smoking; shared input.

These curves show the other-cause (non-lung cancer) mortality for never smokers and for current smokers by smoking quintile (Q, of cigarettes per day) for the male birth cohort of 1950, out to age 99. Former smokers are intermediate to current and never smokers. There is a similar plot for females. These were shared inputs used by all the models. Note that the rates of non-lung cancer mortality represent the US population, not trial (NLST or PLCO) participants.

**Part D. Supplementary Results**

Figure S3. Output from one model showing smoking prevalence by age (calendar year), in a no screening scenario. Proportions of current/former/never smokers are in the presence of lung cancer mortality as well as all-cause mortality.

Figure S4. Output from one model showing smoking prevalence by category of pack-year and age. The proportion of the cohort by age that has accumulated the specified number of pack-years in the presence of lung cancer mortality and other-cause mortality.

Figure S5. Models (no screening) vs. SEER9 and SEER18 incidence, and projections.

For predictions past observed SEER data (over age 60) there are no observed data, but we used an age-period-cohort model to project past observed years (‘Projected’ red double line in plots below), which shows that the models are most divergent after age 85, when SEER data become most sparse. We cannot strictly compare incidence to that in prior birth cohorts since smoking patterns are dissimilar, and incidence varies by cohort.

Figure S6. Mortality in the no-screening scenario, as predicted by the models.

The vertical line at age 90 indicates age at which all event counts (screens, deaths and deaths averted, and life years gained) were truncated for the analyses reported here. Although the models ranked programs similarly, there was variability in the total numbers of predicted lung cancer cases, deaths, and therefore lung cancer deaths prevented. The differences in rates in the no screening scenario in large part explains the predicted differences between models. The four models (E, F, S, and U) which use two-stage or multi-stage clonal expansion models have more similarly shaped curves than the fifth model (M), which does not use a clonal expansion component (see Table S1).


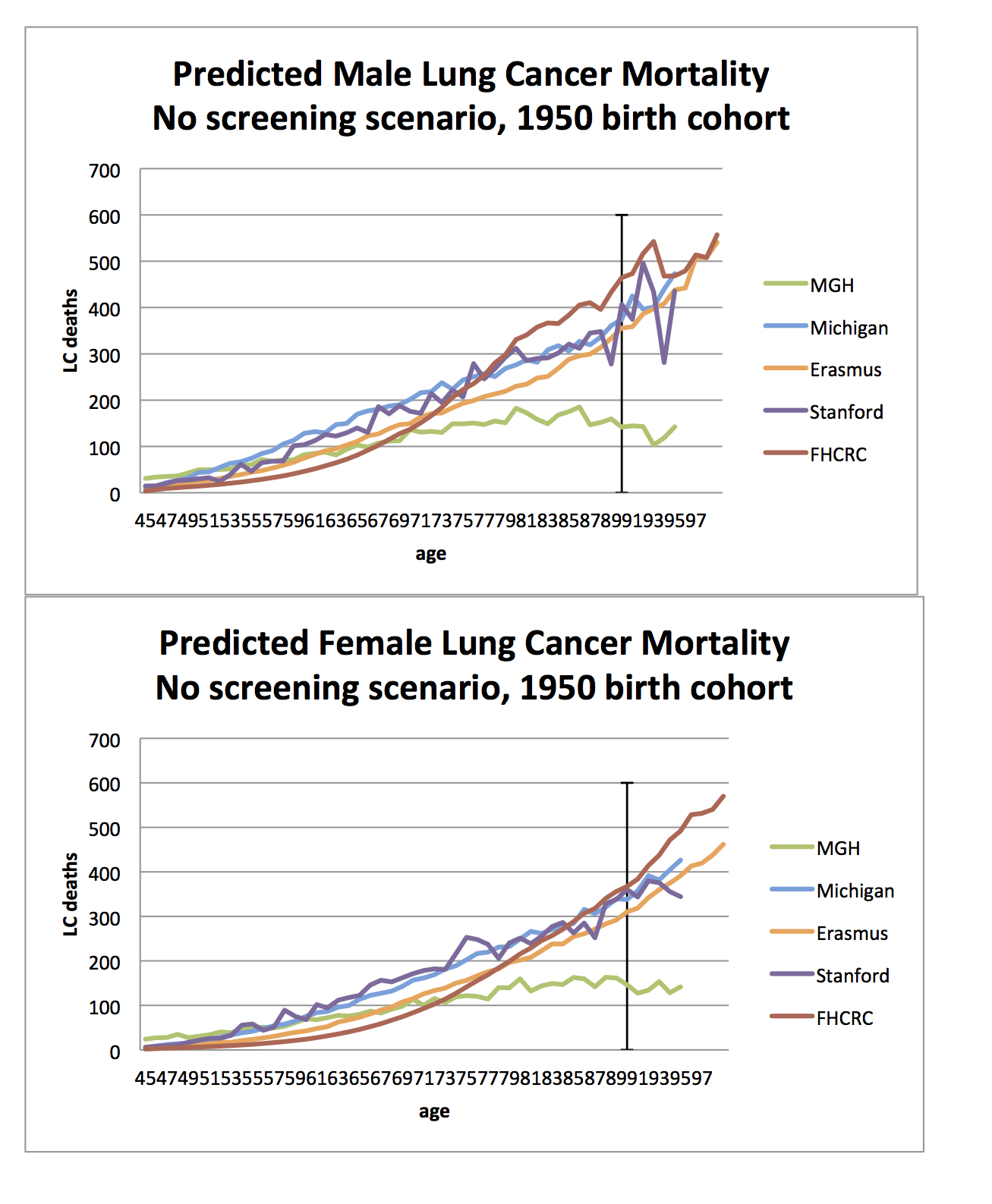


Table S2. Complete list of 120 consensus efficient scenarios, selected as described in the methods. Measure of benefit was lung cancer deaths avoided and measure of harms was number of CT screenings. Table 3 in the main text provides average results across the models for selected scenarios.

A45-80-10-20

A45-80-10-25

A45-80-20-20

A45-80-20-25

A45-80-30-25

A45-85-10-15

A45-85-10-20

A45-85-10-25

A45-85-20-15

A45-85-20-20

A45-85-20-25

A45-85-30-25

A50-80-10-15

A50-80-10-20

A50-80-10-25

A50-80-20-20

A50-80-20-25

A50-80-30-25

A50-85-10-15

A50-85-10-20

A50-85-10-25

A50-85-20-15

A50-85-20-20

A50-85-20-25

A50-85-30-15

A50-85-30-20

A50-85-30-25

A50-85-40-15

A50-85-40-20

A50-85-40-25

A55-80-10-20

A55-80-10-25

A55-80-20-20

A55-80-20-25

A55-80-30-15

A55-80-30-20

A55-80-30-25

A55-80-40-25

A55-85-10-15

A55-85-10-20

A55-85-10-25

A55-85-20-15

A55-85-20-20

A55-85-20-25

A55-85-30-20

A55-85-30-25

A55-85-40-15

A55-85-40-20

A55-85-40-25

A60-80-10-20

A60-80-10-25

A60-80-20-20

A60-80-20-25

A60-80-30-20

A60-80-30-25

A60-80-40-25

A60-85-10-15

A60-85-10-20

A60-85-10-25

A60-85-20-10

A60-85-20-15

A60-85-20-20

A60-85-20-25

A60-85-30-10

A60-85-30-15

A60-85-30-20

A60-85-30-25

A60-85-40-15

A60-85-40-20

A60-85-40-25

B45-85-40-20

B50-80-40-20

B50-80-40-25

B50-85-10-20

B50-85-20-20

B50-85-20-25

B50-85-30-25

B50-85-40-15

B50-85-40-20

B50-85-40-25

B55-80-40-10

B55-80-40-15

B55-80-40-20

B55-80-40-25

B55-85-20-20

B55-85-20-25

B55-85-30-20

B55-85-30-25

B55-85-40-10

B55-85-40-25

B60-80-30-15

B60-80-30-20

B60-80-40-10

B60-80-40-15

B60-80-40-20

B60-80-40-25

B60-85-10-20

B60-85-10-25

B60-85-20-10

B60-85-20-15

B60-85-20-20

B60-85-20-25

B60-85-30-10

B60-85-30-15

B60-85-30-20

B60-85-40-10

B60-85-40-15

B60-85-40-20

B60-85-40-25

T55-85-40-15

T55-85-40-25

T60-75-40-10

T60-80-40-10

T60-80-40-15

T60-80-40-20

T60-80-40-25

T60-85-40-10

T60-85-40-15

T60-85-40-20

T60-85-40-25

Table S3.

| **Result** | **Benefit = LC Deaths Avoided (as in Table S2)** | **Benefit = LYG** |
| --- | --- | --- |
| Number of consensus efficient programs (DEA, 3^rd^ decile) | 120 | 152 |
| Average age start | 54.8 y | 49.5 y |
| Average age stop | 83.2 y | 80.9 y |
| Average minimum pack-year | 27.1 | 27.3 |
| Average years since quit | 19.9 | 17.4 |
| Average frequency | 1.5 y | 1.7 y |

Using life years gained (LYG) as the benefit (versus CT screens performed on the x-axis) yielded a different set of consensus efficient programs. The starting and stopping ages are lower when LYG is maximized, as one might predict due to longer life expectancy among younger individuals. Note that radiation-related risks are not considered in these results.

Figure S7

*
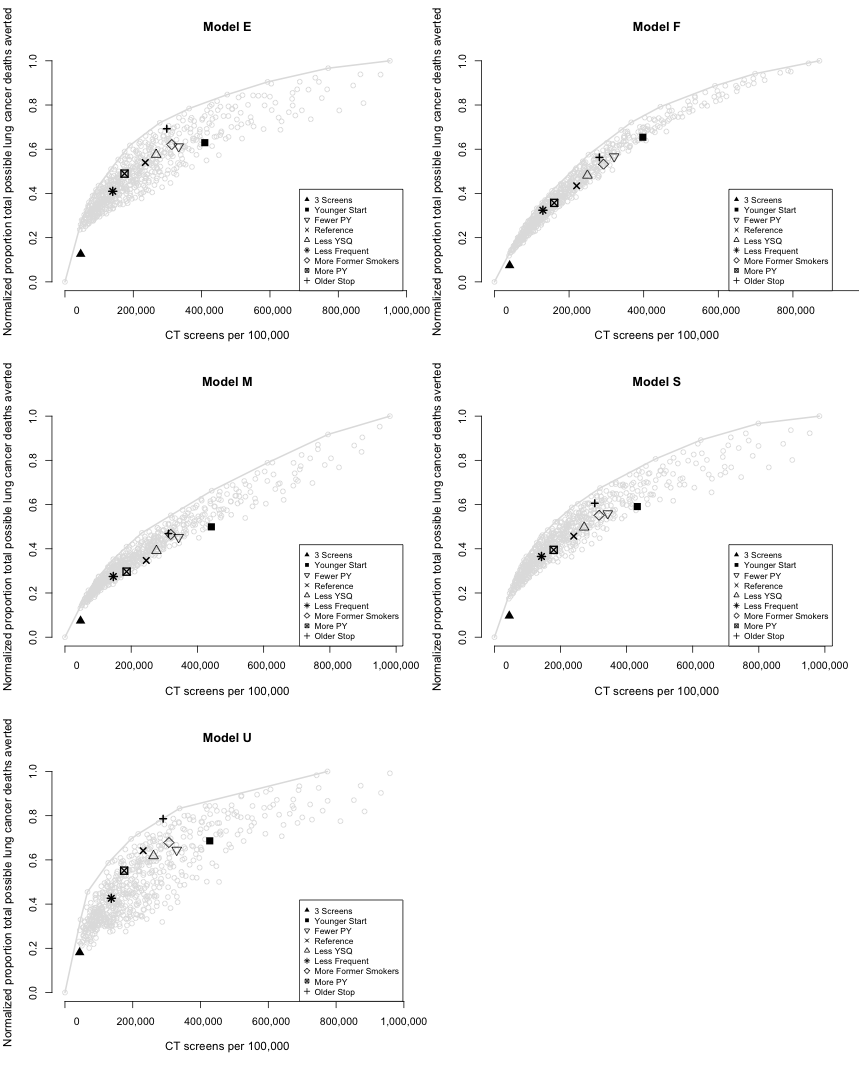
*

Figure S8.


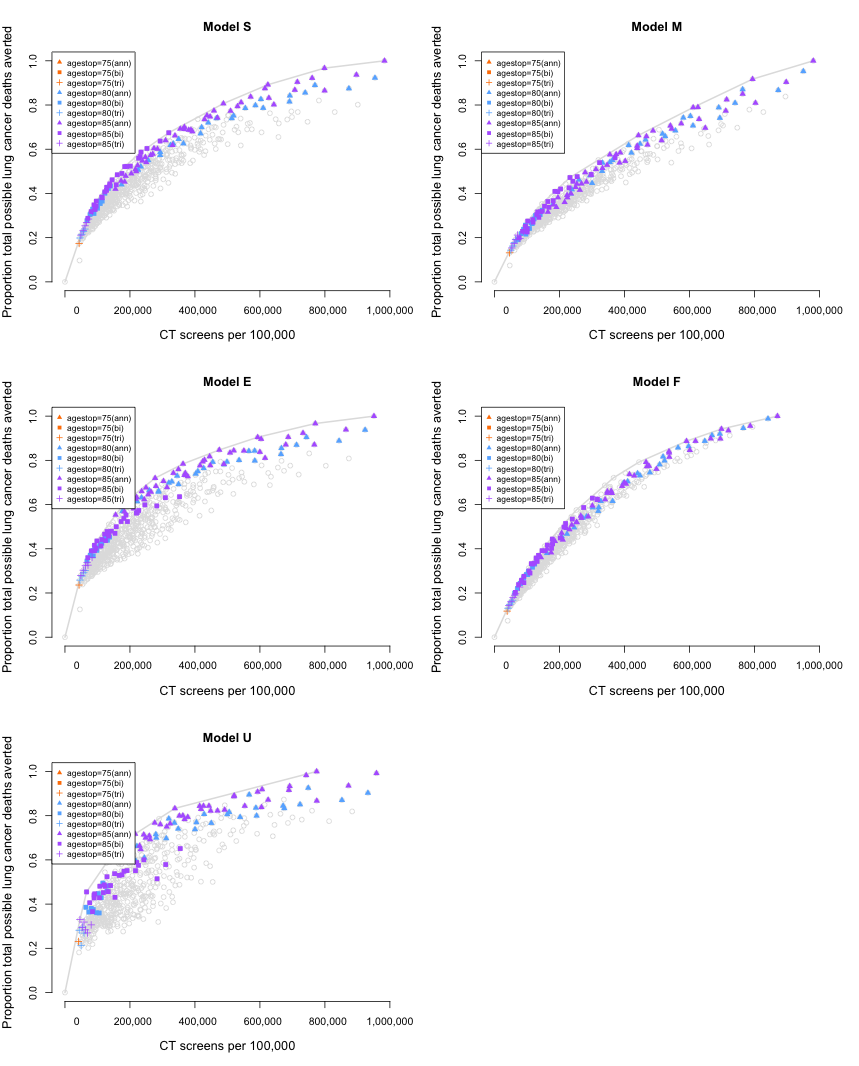


Figure S9

*
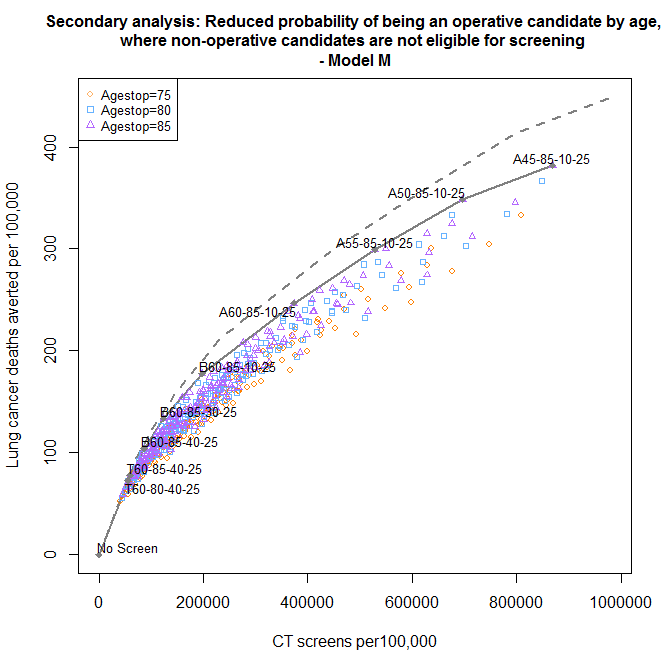
*

The dashed line denotes the efficiency frontier in the main analysis.

**Part E. Supplementary References**

1. Hazelton WD, Jeon J, Meza R, Moolgavkar SH. Chapter 8: The FHCRC Lung Cancer Model. Risk analysis : an official publication of the Society for Risk Analysis 2012;32 Suppl 1:S99-S116.

2. Schultz FW, Boer R, de Koning HJ. Chapter 7: Description of MISCAN-Lung, the Erasmus MC Lung Cancer Microsimulation Model for Evaluating Cancer Control Interventions. Risk analysis : an official publication of the Society for Risk Analysis 2012;32 Suppl 1:S85-98.

3. McMahon PM, Kong CY, Johnson BE, et al. Chapter 9: The MGH-HMS Lung Cancer Policy Model: Tobacco Control versus Screening Risk Analysis 2012;32:S117–S24.

4. McMahon PM, Hazelton WD, Kimmel M, Clarke LC. Chapter 13: CISNET lung models: Comparison of model assumptions and model structures. Risk Analysis 2012;32:S166–S78.

5. McMahon PM, Kong CY, Johnson BE, et al. Estimating long-term effectiveness of lung cancer screening in the Mayo CT screening study. Radiology 2008;248:278-87.

6. Pinsky PF, Gierada DS, Nath PH, Kazerooni E, Amorosa J. National Lung Screening Trial: Variability in Nodule Detection Rates in Chest CT Studies. Radiology 2013.

7. Mery CM, Pappas AN, Bueno R, et al. Similar long-term survival of elderly patients with non-small cell lung cancer treated with lobectomy or wedge resection within the surveillance, epidemiology, and end results database. Chest 2005;128:237-45.

8. Meza R, et al. Calibration of CISNET lung models to NLST and validation with PLCO. Submitted 2013.

9. Meza R, Hazelton WD, Colditz GA, Moolgavkar SH. Analysis of lung cancer incidence in the Nurses' Health and the Health Professionals' Follow-Up Studies using a multistage carcinogenesis model. Cancer causes & control : CCC 2008;19:317-28.

10. Hazelton WD, Goodman G, Rom WN, et al. Longitudinal multistage model for lung cancer incidence, mortality, and CT detected indolent and aggressive cancers. Math Biosci 2012.

11. Moolgavkar SH, Holford TR, Levy DT, et al. Impact of Reduced Tobacco Smoking on Lung Cancer Mortality in the United States During 1975-2000. Journal of the National Cancer Institute 2012.

12. Mandelblatt JS, Cronin KA, Bailey S, et al. Effects of mammography screening under different screening schedules: model estimates of potential benefits and harms. Annals of internal medicine 2009;151:738-47.

13. Zauber AG, Lansdorp-Vogelaar I, Knudsen AB, Wilschut J, van Ballegooijen M, Kuntz KM. Evaluating test strategies for colorectal cancer screening: a decision analysis for the U.S. Preventive Services Task Force. Annals of internal medicine 2008;149:659-69.

14. Draisma G, Etzioni R, Tsodikov A, et al. Lead time and overdiagnosis in prostate-specific antigen screening: importance of methods and context. Journal of the National Cancer Institute 2009;101:374-83.

15. Heijnsdijk EA, Wever EM, Auvinen A, et al. Quality-of-life effects of prostate-specific antigen screening. N Engl J Med 2012;367:595-605. doi: 10.1056/NEJMoa1201637.

16. Tramontano AC, Cipriano LE, Kong CY, et al. Microsimulation model predicts survival benefit of radiofrequency ablation and stereotactic body radiotherapy versus radiotherapy for treating inoperable stage I non-small cell lung cancer. AJR Am J Roentgenol 2013;200:1020-7. doi: 10.2214/AJR.12.8968.

17. McMahon PM, Kong CY, Bouzan C, et al. Cost-Effectiveness of Computed Tomography Screening for Lung Cancer in the United States. Journal of thoracic oncology : official publication of the International Association for the Study of Lung Cancer 2011;6:1841-8.

18. Lin RS, Plevritis SK. Comparing the benefits of screening for breast cancer and lung cancer using a novel natural history model. Cancer causes & control : CCC 2011.

19. Charnes A, Cooper WW, Rhodes E. Measuring the Efficiency of Decision Making Units. European Journal of Operational Research 1978;2:429-44.

20. Oh D, Suh D. nonpareff: Nonparametric Methods for Measuring Efficiency and Productivity. In. 0.5-8 ed: CRAN-R project; 2013.

21. Harris J. Cigarette smoking among successive birth cohorts of men and women in the United States during 1900-80. Journal of the National Cancer Institute 1983;71:473-9.

22. Burns D, Lee L, Shen L, et al. Chapter 2. Cigarette Smoking Behavior in the United States. Washington, DC: National Institutes of Health; 1997. Report No.: NIH Publication No.97-4213.

23. Anderson C, Burns DM, Dodd KW, Feuer EJ. Chapter 2: Birth-cohort-specific estimates of smoking behaviors for the U.S. population. Risk Analysis 2012;32:S14-24.

24. Holford TR. The estimation of age, period and cohort effects for vital rates. Biometrics 1983;39:311-24.

25. Rosenberg MA, Feuer EJ, Yu B, et al. Chapter 3: Cohort Life Tables by Smoking Status, Removing Lung Cancer as a Cause of Death. Risk Analysis 2012;32:S25-S38.
